# Graphene/AlGaN/GaN RF Switch

**DOI:** 10.3390/mi12111343

**Published:** 2021-10-31

**Authors:** Yevhen Yashchyshyn, Paweł Bajurko, Jakub Sobolewski, Pavlo Sai, Aleksandra Przewłoka, Aleksandra Krajewska, Paweł Prystawko, Maksym Dub, Wojciech Knap, Sergey Rumyantsev, Grzegorz Cywiński

**Affiliations:** 1 Institute of Radioelectronics and Multimedia Technology, Warsaw University of Technology, 00-665 Warsaw, Poland; P.Bajurko@ire.pw.edu.pl (P.B.); J.Sobolewski@ire.pw.edu.pl (J.S.); 2 CENTERA Laboratories, Institute of High Pressure Physics PAS, 01-142 Warsaw, Poland; psai@mail.unipress.waw.pl (P.S.); aprzewloka@unipress.waw.pl (A.P.); aleksandrababis@gmail.com (A.K.); maksimdub19f94@gmail.com (M.D.); knap.wojciech@gmail.com (W.K.); roumis4@gmail.com (S.R.); gc@unipress.waw.pl (G.C.); 3 V. E. Lashkaryov Institute of Semiconductor Physics, National Academy of Sciences of Ukraine, 03680 Kyiv, Ukraine; 4 Institute of Optoelectronics, Military University of Technology, 00-908 Warsaw, Poland; 5 Institute of High Pressure Physics PAS, 01-142 Warsaw, Poland; pawel.prystawko@unipress.waw.pl; 6 Laboratoire Charles Coulomb, UMR 5221 CNRS-University of Montpellier, 34950 Montpellier, France

**Keywords:** AlGaN/GaN, graphene, switches, two-dimensional high-density electron gas, millimeter-wave devices

## Abstract

RF switches, which use a combination of graphene and two-dimensional high-density electron gas (2DEG) in the AlGaN/GaN system, were proposed and studied in the frequency band from 10 MHz to 114.5 GHz. The switches were integrated into the coplanar waveguide, which allows them to be used in any system without the use of, e.g., bonding, flip-chip and other technologies and avoiding the matching problems. The on-state insertion losses for the designed switches were measured to range from 7.4 to 19.4 dB, depending on the frequency and switch design. Although, at frequencies above 70 GHz, the switches were less effective, the switching effect was still evident with an approximately 4 dB on–off ratio. The best switches exhibited rise and fall switching times of ~25 ns and ~17 ns, respectively. The use of such a switch can provide up to 20 MHz of bandwidth in time-modulated systems, which is an outstanding result for such systems. The proposed equivalent circuit describes well the switching characteristics and can be used to design switches with required parameters.

## 1. Introduction

Radio frequency (RF) and terahertz dynamically reconfigurable multi-element devices often require electronic switches. Efficient switches are important components for the development of new communication, sensing, imaging, testing, and instrumentation systems. Applications of such switches include switched-beam reconfigurable antennas, polarization switching, multi-band receivers, transceivers, time division duplexing systems, and test circuits with multiple signal paths.

There are a number of technologies available for millimeter-wave switches. Most of them employ transistors based on typical semiconductor manufacturing technologies using silicon or III–V semiconductors [1,2,3,4,5,6,7,8,9,10,11]. Besides these, there are less conventional technologies available, such as micro-electro-mechanical systems (MEMS) [12,13] and switches based on phase changing materials (PCM) [14,15].

Graphene and transition metal dichalcogenides also attract attention for designing RF and terahertz switches and other devices [16,17]. A monolayer MoS_2_ RF switch with a 0–50 GHz operating frequency range was reported in [18]. Several publications consider the possibilities of graphene-based RF switches for the millimeter-wave band [19,20,21,22]. However, these papers include only theoretical analysis and/or computer simulations.

Graphene can be also used to construct nanoelectro-mechanical systems (NEMS). This concept was investigated in [23,24,25]. The switches were based on a graphene membrane suspended over a specially constructed coplanar waveguide (CPW). When a bias voltage was applied, the membrane bent and created a short circuit in the waveguide.

Considering variable attenuators as a specific kind of switch, one can notice more examples of graphene-based devices. In [26], a prototype of a controllable attenuator based on graphene integrated with an antenna system was designed, fabricated, and measured. The attenuator allows beam steering at mm-wave frequencies. In [27], a novel tunable grounded CPW attenuator based on graphene nanoplates was also proposed. Further examples of graphene-based CPW and microstrip line attenuators can be found in [28,29], respectively.

Graphene-based modulators should be also mentioned, since they can be based on switches [30,31,32,33].

What is common in the abovementioned publications on graphene-based switches is that graphene is used as an active switching element: a change in the graphene properties under bias allows the RF power to be switched.

In this paper, we propose and study an approach wherein the combination of graphene and two-dimensional high-density electron gas (2DEG) in the AlGaN/GaN system allows us to switch effectively at RF and mm-wave frequencies. The switch was integrated into the planar transmission line. A CPW was chosen because it has smaller losses in comparison with the microstrip line. This approach allowed us to avoid the problems with packaging and interconnects, which can cause high losses, especially at mm waves.

## 2. Structure of Graphene/AlGaN/GaN Switch and DC Characteristics

**Figure 1:**
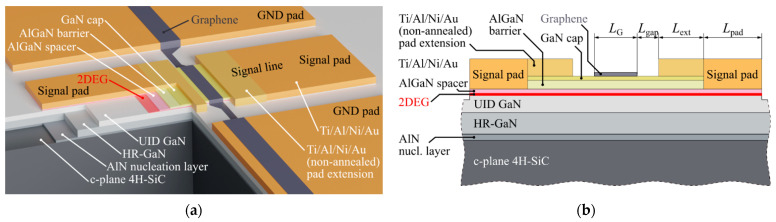
(**a**) Cutaway view of the structure; (**b**) cross-section along the constructed coplanar waveguide (CPW).

The proposed hybrid structure includes a CPW with an embedded graphene/AlGaN/GaN switch. A typical CPW consists of two ground conductors and a signal conductor line in the middle. In the proposed structure, the signal conductor line is made of Ti/Al/Ni/Au on the top of the AlGaN/GaN structure. The signal line is interrupted in the middle and the graphene gate device is placed in the gap between two parts of the signal line (see Figure 1). In the on state, the connection between two parts of the signal line is provided by 2DEG. The graphene layer in the middle of the device, above 2DEG, acts as a gate. The graphene gate is connected to the ground (GND) of the CPW; therefore, the device is controlled by the bias voltage applied to the CPW signal pads along with a high-frequency signal. As a result, the structure operates as a high-frequency single pole, single throw (SPST) switch.

As opposed to a conventional transistor-based design, where a metal gate is used, the properties of a graphene gate also change when the voltage is applied between the graphene gate and 2DEG. Therefore, the properties of both the 2DEG and graphene are controlled by the bias. In other words, the 2DEG also can be considered as a “gate” relative to graphene. Since CVD graphene is of p-type conductivity, a positive voltage on the 2DEG relative to graphene reduces the concentration of holes and increases the graphene resistivity [34]. Therefore, with properly designed graphene and two-dimensional electron gas at the AlGaN/GaN interface, a positive voltage on the 2DEG relative to graphene can completely remove the conductive layers in the central line gap of the CPW. This should improve the off-state characteristics.

We used AlGaN/GaN epitaxial heterostructures grown by Metalorganic Vapor Phase Epitaxy (MOVPE) on a silicon carbide substrate. MOVPE growth was started from a 38-nm-thick AlN nucleation layer on a commercially available 500-µm-thick semi-insulating SiC substrate. The next layer was 2.3 µm high-resistivity (HR) GaN buffer followed by a 0.7 µm unintentionally doped (UID) GaN layer. The AlGaN barrier consisted of: 1.2 nm Al*_x_*Ga_1−*x*_N (*x* = 66%), 5 nm Al*_x_*Ga_1−*x*_N UID (*x* = 28%), 10 nm AlGaN:Si (n~1.5 × 10^18^ cm^−3^), and a 2 nm UID AlGaN layer. The whole heterostructure was covered by a 2 nm GaN cap layer. A schematic diagram of the fabricated heterostructures is shown in Figure 1. This is a typical structure of AlGaN/GaN high electron mobility transistors (HEMTs) for high-frequency applications [32].

The CPW processing was performed using a commercial laser writer system for lithography based on a 405 nm wavelength GaN laser source with a minimum 1 µm linewidth. The first step in the processing was 150 nm mesa etching provided by an Inductively Coupled Plasma–Reactive Ion Etching system. As a result of the etching, the 2DEG remained only under the signal line. Then, ohmic contacts were formed by thermal evaporation of Ti(15 nm)/Al(100 nm)/Ni(40 nm)/Au(50 nm) and rapid thermal annealing at 780 °C for 1 min under a N_2_ atmosphere. Metallization for the ohmic contacts was deposited on the central line and interrupted in the middle. The ground lines were fabricated simultaneously. In order to be able to tune the width of the gap in the central line, the same Ti/Al/Ni/Au metal stack may be deposited at a later time in order to reduce the gap width. This metal stack was not annealed.

The last step was graphene transferring and its patterning. A chemical vapor deposition (CVD) graphene layer was deposited on the whole GaN-based wafer by the high-speed electrochemical delamination technique [35]. The detailed step-by-step procedure of the graphene delamination and transferring from Cu foil onto AlGaN/GaN can be found in [36]. Finally, graphene patterning was performed by oxygen plasma etching. Graphene remained in the central line gap and was extended to the ground pads as shown in Figure 1a. A cross-section of the structure is shown in Figure 1b.

**Figure 2:**
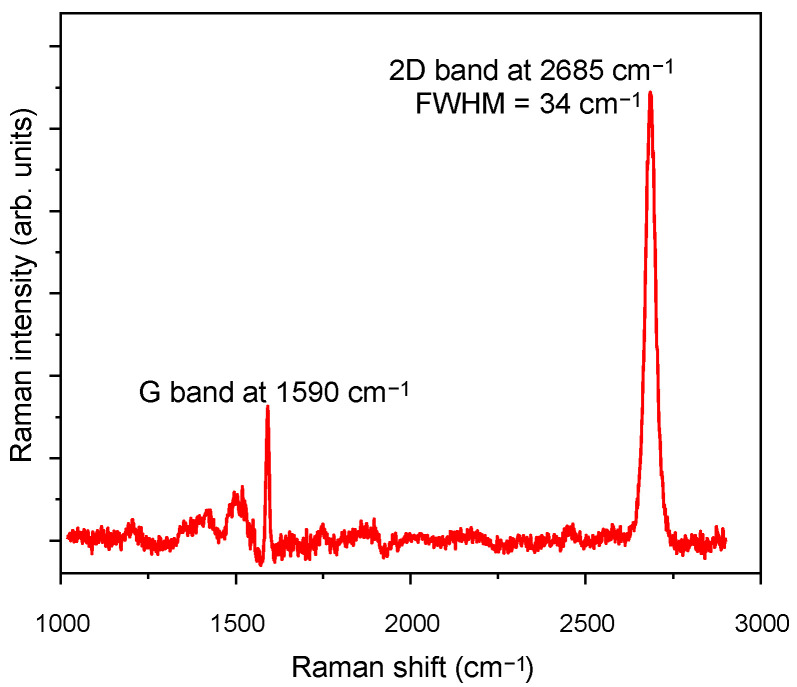
Raman spectrum of the graphene layer on the AlGaN/GaN wafer grown on SiC substrate.

During the CPW fabrication, the quality of the graphene layer was controlled with Raman spectroscopy. Figure 2 shows the Raman spectra of graphene transferred onto the AlGaN/GaN wafer, recorded with a Renishaw inVia micro-Raman system using a 532 nm frequency doubled Nd:YAG laser as an excitation source. The typical graphene peaks were observed: G mode at 1590 cm^−1^ and 2D mode at 2685 cm^−1^, which are characteristic of the sp^2^ hybridization of carbon. The full width at half maximum (FWHM) of the 2D band and the intensity ratio of the 2D/G peaks were used to determine the number of graphene layers and their quality. The FWHM of the 2D peak in Figure 2 was ~34 cm^−1^, which is typical for monolayer graphene. The ratio I_2D_/I_G_ was over 2, which is characteristic of a monolayer of graphene as well. The obtained results confirmed that the graphene sample was of high quality and defect-free. The spectrum in the range from 1050 cm^−1^ to 1950 cm^−1^ showed also features stemming from the SiC substrate.

**Figure 3:**
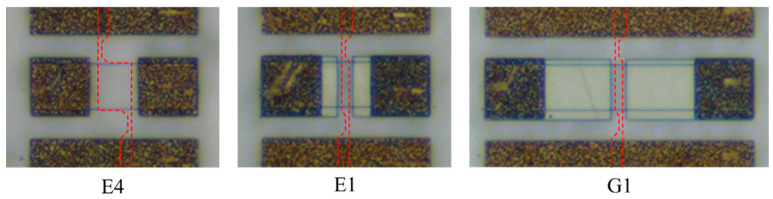
Fabricated graphene/AlGaN/GaN RF switches.

The optical microscope images of three of the fabricated devices are shown in Figure 3. Since graphene is barely seen in an optical microscope, its location is outlined with red dashed lines. The dark color corresponds to the annealed contact metallization, and the lighter color is the metallization deposited at the second step. The dimensions of the structures are summarized in Table 1.

**Figure 4:**
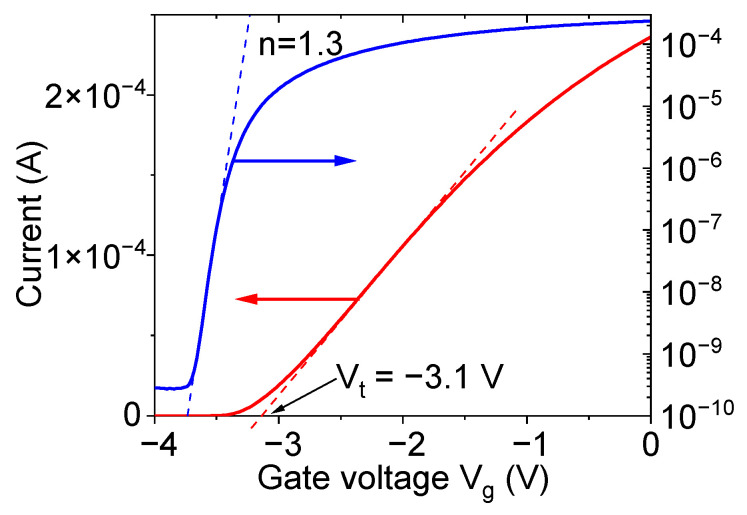
Transfer current–voltage characteristics of the transistor in E4 structure. Drain voltage V_d_ = 0.1 V.

The structures shown in Figure 3 represent the field effect transistors with the left and right sides of the central line acting as the source and drain. The graphene layer in the middle, which is located between the source and drain on the top of the AlGaN barrier layer, acts as a gate. It was shown previously [36] that graphene forms a high-quality Schottky barrier to AlGaN and graphene gate AlGaN/GaN transistors demonstrate very good characteristics. Figure 4 shows the transfer current–voltage characteristics of the transistor in the E4 structure. The transistors demonstrated around six orders of magnitude for the on–off ratio and the subthreshold slope *n* = 1.3–1.4. The subthreshold current, which is determined by the gate leakage current, was very small, even for the transistor with the highest gate area, as shown in Figure 4. The current–voltage characteristic shown in the linear scale in Figure 4 only slightly tends to saturate at high gate voltages, indicating the minor contribution of the contact resistance. The threshold voltage for the studied transistors determined from the linear extrapolation of the current–voltage characteristic at a small drain voltage was within the range of V_t_ = −3.0 to −3.2 V. This means that at V_g_ < −3.5 V, the channel is fully depleted and the transmission line is interrupted. It is connected only due to the highly resistive graphene layer and fringing capacitances. At zero gate voltage, the central line is connected by the highly conductive 2DEG.

## 3. RF Characteristics

**Figure 5:**
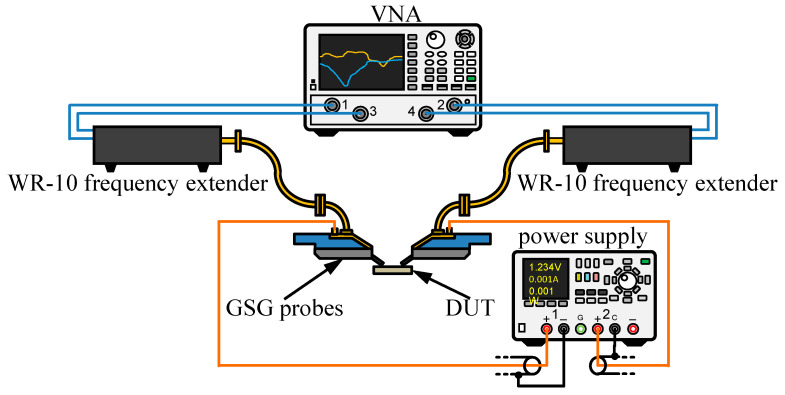
Schematic diagram of the measurement setup (DUT stands for “device under test”).

**Figure 6:**
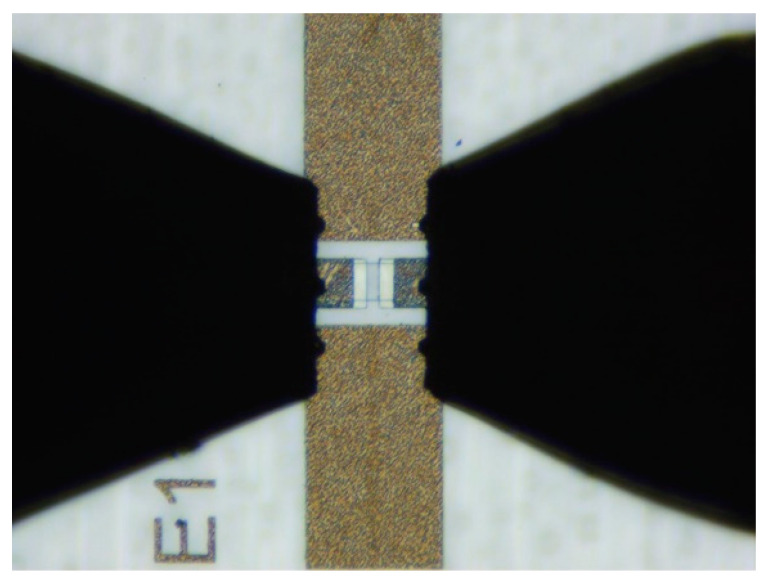
Microscopic view of graphene/AlGaN/GaN switch with probes attached.

On-chip S-parameter measurements of the graphene/AlGaN/GaN switches in the 70.5–114.5 GHz frequency range were carried out using a measurement setup configured as shown in Figure 5. The setup consisted of the Agilent N5245A PNA-X vector network analyzer (VNA) with VDI WR-10 waveguide frequency extenders and 100 µm pitch Cascade Microtech Infinity WR-10 waveguide GSG probes. The probes were positioned using the Cascade Microtech EPS200MMW probe station. For the measurements at frequencies below 50 GHz, the same setup was used but without frequency extenders and with coaxial GSG probes. Figure 6 shows an optical microscope image of the structure under testing with the probes attached.

For the calibration and setting of the measurement plane to the contact pads, the SOLR Cascade 101-190C and LRM Cascade 138-357 standard impedance substrates were used at low and high frequencies, respectively. The S-parameter measurements were performed with bias voltages applied between the central lines and ground. The DC connections were provided through the bias ports of the VNA and through the bias ports of the waveguide probes in the low- and high-frequency configurations, respectively. As the graphene layer was connected to the GND conductors (Figure 1a), a positive voltage applied to the signal pads corresponded to the negative bias of the graphene gate.

**Figure 7:**
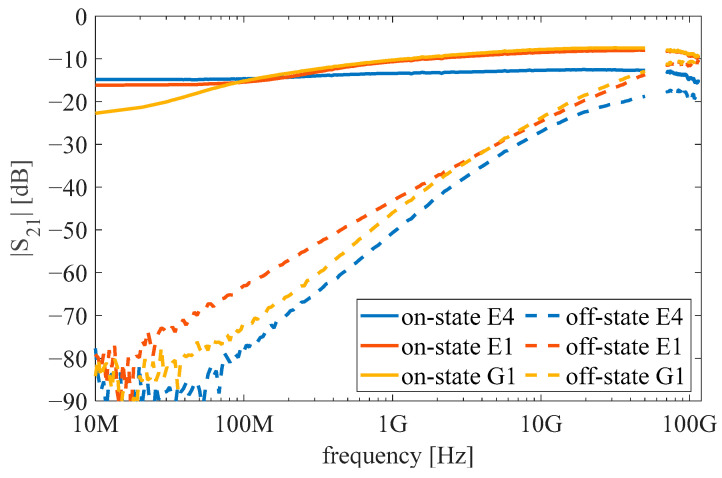
Measured transmission coefficient characteristics of the graphene/AlGaN/GaN switches.

Figure 7 shows the measured transmission coefficient S21 characteristics in the on and off state of three examined graphene/AlGaN/GaN switches (see Table 1 for details). In the studied configuration, the on state corresponds to zero gate voltage and the off state was studied at V_g_ = −5 V.

The structure E4 with the large gap in the central line *L* = 2*L*_gap_ + *L*_g_ = 65 μm exhibits insertion loss between 12.5 and 14.9 dB in the on state across a very wide frequency range. On the other hand, the off-state characteristics show the strong frequency dependence of isolation, which is typical for parasitic capacitive coupling. Isolation values decrease from over 77 dB below 100 MHz to 17.5–19.4 dB in the 70.5–114.5 GHz frequency range. Despite poor isolation at frequencies above 70 GHz, the switching effect is still evident, with an on–off ratio of approximately 4 dB.

Narrowing the gap in the central line to *L* = 2*L*_gap_ + *L*_g_ = 20 μm in the E1 and G1 structures allowed us to achieve lower on-state insertion loss (7.4–14.1 dB) at frequencies above 200 MHz. A smaller gap between extended pads has an adverse impact on the isolation due to increased capacitive coupling between the input and output. As a result, at frequencies above ~100 GHz, switching is not effective. E1 and G1 structures have different dimensions of extension pads, which causes their slightly different behavior at low frequencies.

In order to study the switching properties in the time domain, the DC power supplier was replaced with the function generator.

**Figure 8:**
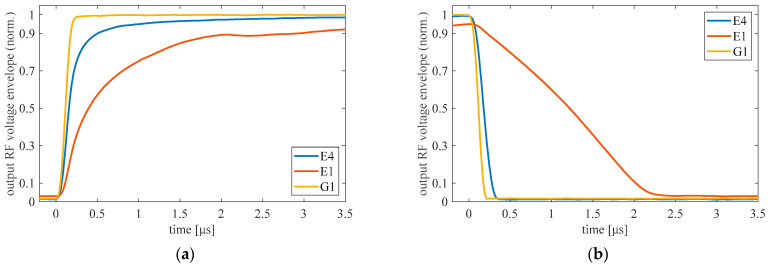
Turn-on (**a**) and turn-off (**b**) transient behavior of output RF voltage envelope of the graphene/AlGaN/GaN switches measured at 1 GHz. The waveforms are normalized to their own amplitude in steady on state.

Figure 8 shows the switching dynamics of a 1 GHz signal at the output. The waveforms presented in Figure 8 were obtained with 100 kHz, with a 5 V square wave signal applied to the structures.

The measured switching times are listed in Table 2. The large differences in switching times between structures can be attributed to technological uncertainties, particularly to the graphene quality. The switch based on the G1 structure exhibits a very fast switching time. The rise and fall times are 25 ns and 17 ns, respectively. The use of such a switch can provide up to 20 MHz of bandwidth in time-modulated systems, which is an outstanding result for such systems [10,37].

**Figure 9:**
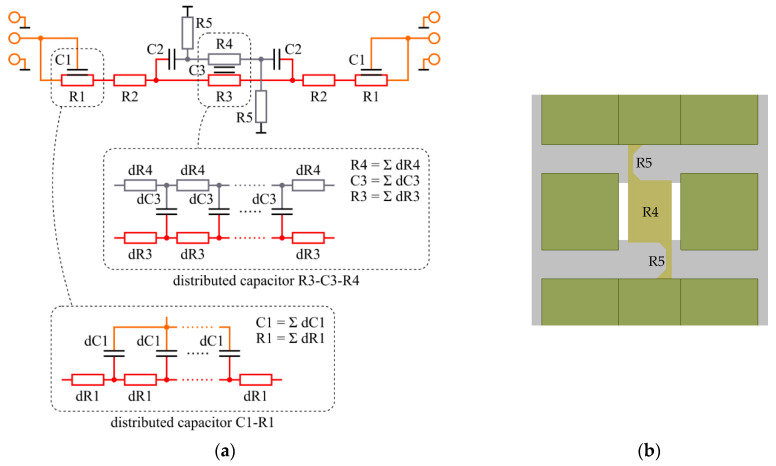
(**a**) Equivalent small-signal circuit for both on and off states. Colors refer to the physical structure: red—2DEG, orange—contact pads and its extensions, gray—graphene. (**b**) Schematic view of the switch with indicated resistances of graphene, R4, and R5.

The measured transmission characteristics shown in Figure 7 were compared with the characteristics of an equivalent small-signal circuit of the structures shown in Figure 9a. The circuit represents the physical design of the structure.

The graphene gate is represented by resistors R5-R4-R5 arranged according to the gate shape (Figure 9b). The graphene gate and 2DEG underneath constitute a capacitor with resistive plates, which is represented as an element R3-C3-R4. This element is actually a distributed capacitor and it is modeled as an infinite number of elementary stages dR3-dC3-dR4, connected as shown in Figure 9a. The values of R3-C3-R4 elements are the sums of their elemental counterparts. Two capacitors C2 represent the fringe capacitances of the graphene gate (mainly responsible for limited isolation in the off state).

Similarly, pad extension with 2DEG underneath constitutes a capacitor with a single resistive plate R1-C1. Values of circuit elements are given in Table 3. They were extracted from nominal structure parameters and DC current–voltage characteristics, except for C2, which was matched based on the measured data.

**Figure 10:**
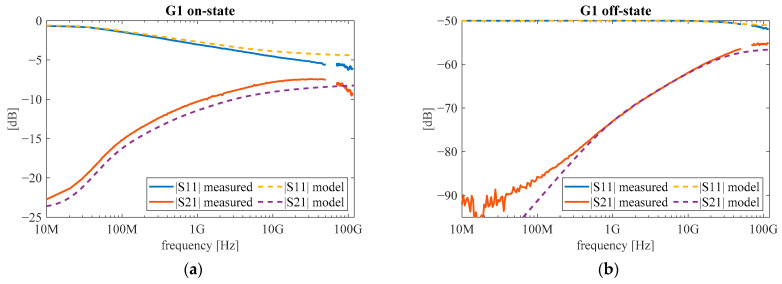
S11 and S21 scattering parameters for the G1 structure calculated from equivalent small-signal circuit along with measurement results for (**a**) on and (**b**) off states.

The comparison of measurements and simulations of the S11 and S21 parameters for the G1 structure is presented in Figure 10. For both reflectivity (S11) and transmission (S21), the simulation corresponds to the measurement in both operating states. Simulations of the E4 and E1 structures also showed good agreement with the measurements.

The results of simulations shown in Figure 10 indicate that the proposed equivalent circuit represents well the behavior of the studied graphene switches and can be used for the designing of switches with required parameters.

## 4. Discussion

Table 4 compares the parameters of the studied switches with previously published results for graphene-based RF switches. As can be seen, the graphene/AlGaN/GaN switches demonstrate very good characteristics, in many ways better than other published simulated and experimental results. The proposed devices operate with a low driving voltage and very low power consumption, providing very fast switching. As seen in Table 4, this kind of graphene-based switch is one of the very few switches whose parameters have been studied experimentally.

Although the switches in more conventional technologies demonstrate better high-frequency performance, this research is in the early stage and there is room for improvement. The advantage of the application of graphene as a gate in a millimeter-wave switch is that its parameters change with bias at the same time as the channel parameters. Therefore, in the off state, with positive bias on the 2DEG relative to the graphene gate, the conductive layers can be eliminated from the gap in the CPW, making the impact of parasitic connection through fringing capacitances and the gate less significant. The main directions of the future development of this type of structure are the optimization of graphene characteristics and switch geometry as well as the evaluation of shunt architecture to achieve lower insertion losses and a higher operating frequency band.

## 5. Conclusions

We have proposed and studied the design of a switch that uses a combination of graphene and two-dimensional high-density electron gas (2DEG) in an AlGaN/GaN system in order to provide effective switching at RF frequencies. The switch was integrated into the coplanar waveguide, which was chosen due to lower losses compared to the microstrip line, especially at high frequencies. The presented design is an on-chip solution fabricated in one technological process. The proposed equivalent circuit describes well the switching characteristics and can be used to design switches with required parameters. The switching times are sufficiently low to use this kind of switch in time-modulated systems.

## Figures and Tables

**Table 1:** Dimensions of the graphene/AlGaN/GaN switches.

Structure	*L*_G_	*L*_gap_	*L*_ext_	*L*_pad_
E4	45 μm	10 μm	0 μm	80 μm
E1	10 μm	5 μm	22.5 μm	80 μm
G1	10 μm	5 μm	90 μm	80 μm

**Table 2:** Measured switching times (between 10% and 90% of the RF voltage).

Structure	Rise Time	Fall Time
E4	360 ns	120 ns
E1	2900 ns	1750 ns
G1	25 ns	17 ns

**Table 3:** Equivalent schematic element values extracted from nominal structure parameters and DC characteristics.

Structure	State	R1	C1	R2	R3	C2	C3	R4	R5
E4	ON	0 Ω	0 pF	50 Ω	320 Ω	16 fF	11.8 pF	1 kΩ	8 kΩ
	OFF				∞				
E1	ON	160 Ω	5.9 pF	45 Ω	72 Ω	22 fF	2.6 pF	233 Ω	8 MΩ
	OFF				∞				
G1	ON	645 Ω	24 pF	45 Ω	72 Ω	22 fF	2.6 pF	233 Ω	8 kΩ
	OFF				∞				

**Table 4:** State of the art for graphene-based RF switches.

Ref.	Configuration	Max. Freq. (GHz)	Insertion Loss (dB)	Isolation (dB)	Rise/Fall Time (ns)	Control Voltage (V)	Driving Current (mA)	Validation of the Results
[19]	graphene shunt with graphene gate	60.8	1.1	34.5	n/a	33	~0 (isolated gate)	simulation study
[20]	graphene shunt	70	5	>33	n/a	30	n/a	simulation study
[23,24]	graphene NEMS shunt	60	0.2	>20	n/a	7	~0 (isolated membrane)	simulation study
[25]	graphene NEMS shunt	110	1.2	<15	n/a	n/a	~0 (isolated membrane)	simulation study
[27]	graphene shunt (attenuator)	28	2.5	14	n/a	6	65	measurement
[28]	graphene shunt (attenuator)	40	3	11.5–15	n/a	4	n/a	measurement
This work	2DEG series circuit with graphene gate	84 ^1^	7.4–19.4	17.5–77	25/17	5	~0 (isolated gate)	measurement

^1^ The max. frequency is calculated as cut-off frequency *f*_cut-off_ = 1/(2π·R_on_·C_off_), where C_off_ = ½·C2, R_on_ = R3 + 2·R2 + 2·R(R1-C1), R(R1-C1) is the resistance of R1-C1 circuit at cut-off frequency. For the E1 and G1 structures, R(R1-C1) ≈ 5 Ω at 84 GHz. The values of C2, R2, and R3 are given in Table 3. For the E4 structure, *f*_cut-off_ = 47 GHz.
